# Analyzing the effect of sibling number on input and output in the first 18 months

**DOI:** 10.1111/infa.12578

**Published:** 2024-01-06

**Authors:** Catherine Laing, Elika Bergelson

**Affiliations:** 1 University of York, York, UK; 2 Harvard University, Cambridge, Massachusetts, USA

## Abstract

Prior research suggests that across a wide range of cognitive, educational, and health-based measures, first-born children outperform their later-born peers. Expanding on this literature using naturalistic home-recorded data and parental vocabulary reports, we find that early language outcomes vary by number of siblings in a sample of 43 English-learning U.S. children from mid-to-high socioeconomic status homes. More specifically, we find that children in our sample with two or more—but not one—older siblings had smaller productive vocabularies at 18 months, and heard less input from caregivers across several measures than their peers with less than two siblings. We discuss implications regarding what infants experience and learn across a range of family sizes in infancy.

## 1 | INTRODUCTION

A common simplifying assumption in research on language development is that there is a theoretical “optimum” environment for early language, whereby the input is tailored to a single infant’s needs, changing over time as language capacity grows (Soderstrom, 2007; Stern et al., 1983). However, for many infants and for many reasons, language acquisition occurs across diverse social contexts that can influence the learning environment, including the presence of older siblings in the home (Fenson et al., 1994). According to the United States Census Bureau (2010), around one third of children are born into households with at least one other infant present, and one in every five infants is acquiring language in a household shared with two or more other children. Similar statistics are reported for British infants (Office for National Statistics, 2018), where the average household has 1.75 children, and 15% of households have three children or more. More globally, in most parts of the world, few children grow up without siblings (United Nations, 2017). In this paper, we consider the role of siblings in the early language environment of English-learning infants. We use naturalistic home-recorded data to measure input in earlier- and later-born infants, alongside their productive vocabulary over the first 18 months of life.

Prior research suggests that infants born to households with older children may be slower to learn language. Fenson et al. (1994) found that by 30 months of age, children with older siblings performed worse than those with no siblings across parent-reported measures of productive vocabulary, use of word combinations, and mean length of utterance. This “sibling effect” may be the result of differences in input between first- and later-born children: some research finds that infants with older siblings hear less speech aimed specifically at them, and what they do hear is understood to be linguistically less supportive of early language development (Hoff-Ginsberg, 1998; Oshima-Takane & Robbins, 2003). In contrast, some studies have noted linguistic *advantages* for later-borns, who may have stronger social-communicative skills (Hoff, 2006), better understanding of pronouns (Oshima-Takane et al., 1996), and better conversational abilities (Dunn & Shatz, 1989). Overall, while the particulars differ across studies, prior work suggests that the presence of siblings in the home leads to differences in infants’ early linguistic experiences and skills, though the direction of these differences varies depending on what aspects of language are being measured.

Numerous studies have attempted to better understand how siblings affect the language development trajectory, with comparisons of language acquisition across first- and later-borns. Here again, findings are mixed, but overall two general conclusions can be drawn. First, analyses consistently show that infants with older siblings generally have slower *vocabulary development* (Berglund et al., 2005; Fenson et al., 1994; Pine, 1995; Zambrana et al., 2012), and this effect increases with number of older siblings (Gurgand et al., 2022; Karwath et al., 2014; Peyre et al., 2016). Furthermore, this finding is consistent across cultures (e.g., European French (Gurgand et al., 2022; Havron et al., 2019); Singaporean (Havron et al., 2022); Kenyan (Jakiela et al., 2020); and German (Karwath et al., 2014)). However, this finding is not as clear-cut as has been previously assumed: Hoff-Ginsberg (1998) shows first-borns to have better lexical and syntactic skills up until 2;5, but later-born infants had better conversational abilities during the same time-period. Recent studies have also identified effects for age gap between the target child and their siblings (whereby larger age gaps correlate with lower vocabulary scores, Gurgand et al., 2022; Havron et al., 2022) and sibling sex (whereby older brothers have a negative effect on vocabulary outcomes, but not older sisters, Havron et al., 2019; Jakiela et al., 2020) though neither of these effects are found consistently across datasets; Havron et al. (2022) and Gurgand et al. (2022) find no effect for sibling sex, whereas Havron et al. (2019) find no effect for age gap. Some of these differences across studies may relate to insufficient power to detect relatively small effects, perhaps leading to under- or over-estimation of effect sizes, or simultaneous contributing factors that are difficult to disentangle.

The second general finding pertains to sibling-related differences in the early linguistic *environment*: infants with no siblings receive more input overall, and this more closely reflects what is typically considered to be “high quality” input in the extant literature (i.e., more input in an infant-directed speech style (Ramírez-Esparza et al., 2014); longer utterance length (Barnes et al., 1983); higher lexical diversity (Rowe & Snow, 2020)). Indeed, the very presence of a sibling in the linguistic environment changes the way language is used. When siblings are present (i.e., triadic interactions), mothers’ input has been found to be more focused on regulating behavior, as opposed to the language-focused speech that is common in dyadic contexts (Oshima-Takane & Robbins, 2003). Reports show that the mean length of utterance is longer in the input of first-born infants (Hoff-Ginsberg, 1998; but see also Oshima-Takane & Robbins, 2003 for a comparison of dyadic and triadic contexts), who also hear more questions directed at them than later-borns. Both Jones and Adamson (1987) and Oshima-Takane and Robbins (2003) report no difference between the overall number of word types produced by mothers in dyadic and triadic settings, but the proportion of speech directed at the target infant is drastically reduced when input is shared with siblings.

As Hoff (2006) explains, infants with siblings have less experience of speech directed at them, but they do have an advantage over their first-born peers in that they are subject to more overheard speech. This may be an important source of input for infants with one or more older siblings. Akhtar et al. (2001) show that, by age 2;6, children can learn both novel object labels and novel verbs through overhearing. Slightly younger children (aged 1;11–2;2) were also able to learn the novel object labels, but not verb labels. Two-year-old infants can even learn novel object labels while doing activities that distract them from the language input, and when the novel words are produced non-saliently (Akhtar, 2005). This suggests that, while the learning environment for later-borns might differ from that of first-born infants, there may be ample opportunity for them to learn from the speech that surrounds them; namely overheard speech directed at their older sibling(s). Evidence is mainly drawn from work testing infants aged 2 and above (e.g., Akhtar, 2005; Fitch et al., 2020; Foushee et al., 2021), and generally relies on experimental work rather than observations of the home environment. However, Floor and Akhtar (2006) tested younger infants to find that the capacity to learn from overheard speech is available from as early as 16 months, at least in an experimental setting.

There thus may be a trade-off, even in early development, between highly supportive one-to-one input from a caregiver (cf. Ramírez-Esparza et al., 2014) and the potential benefits drawn from communicating with (or overhearing communication with) a sibling. In the present study, we test the extent to which having more versus fewer siblings in the home environment may affect the linguistic environment in ways that could lead to differences in vocabulary development over the course of the first 18 months of life. In analyzing infants’ growing productive vocabulary and linguistic environment in relation to the presence of older siblings in their household, the present work expands on the extant literature in two key ways. First, much of the existing literature identifying links between sibling number and vocabulary outcomes draws on large-scale questionnaire data, rather than naturalistic day-to-day interactions in the home. In contrast, we analyze an existing corpus of home recordings in concert with vocabulary checklists, in order to capture the reality of the early linguistic environment and how this is affected by sibling number. Second, we consider the opportunities that overheard speech might present in the infant’s linguistic environment. We examine the effect of sibling number on overall amount of input produced in our naturalistic recordings, as well as, crucially, the extent to which parents label objects being attended to by the infant (*object presence*^1^). The analysis of object presence will allow us to gain insight into the kinds of learning opportunities being presented to infants in the early input, based on the previous research showing that object labeling—even when not directed specifically at the target child—can be a valuable source for acquiring linguistic knowledge. Based on work summarized above, we expect that both the language environment and infants’ early productive vocabulary will vary as a function of how many older siblings they have.

### 1.1 | Hypotheses

Synthesizing the work above in broad strokes, given prior research showing that early lexical development is more advanced among first-born infants (Hoff-Ginsberg, 1998), we predict that children with more siblings will have lower productive vocabularies than their peers with fewer siblings. However, we have no a priori predictions about how these differences will manifest gradiently (e.g., linear decrease for each additional sibling, a threshold effect where we see a drop after a certain sibship size, etc.).

With regard to the infants’ linguistic environment, we hypothesize that infants with more siblings will experience lower prevalence of two aspects of the language input previously shown to support language development: **amount of input** and **amount of object presence**. Just as for productive vocabulary size, we do not make a priori predictions regarding the shape of these effects, beyond predicting a decrease with sibling number. Regarding input specifically, following previous studies that show infants with siblings to receive less speech directed at them (Jones & Adamson, 1987; Oshima-Takane & Robbins, 2003), we expect to see the same effect in our sample. In terms of object presence (by which, as noted above, we mean word and object co-occurrence, e.g., mother saying “cat” when the child is looking at a cat), we predict a decrease as sibling number increases. This is because, by hypothesis, as caregivers’ attention is drawn away from one-to-one interactions with the infant, there is likely less opportunity for contingent talk and joint attention. Prior research suggests links between object presence and early word learning (Bergelson & Aslin, 2017; Cartmill et al., 2013), though to our knowledge this has not been examined in relation to sibling number.

## 2 | METHODS

We analyze data from the SEEDLingS corpus, a longitudinal set of data incorporating home recordings, parental reports and experimental studies from the ages of 0;6 to 1;6. See Bergelson et al. (2019) for further details on the full set of home-recorded data and its annotations. The present study draws on the parental report data to index child vocabulary size, and annotations of hour-long home video recordings, taken on a monthly basis during data collection, to index input.^2^ We note at the outset that with such a multidimensional dataset there are always alternative ways of conducting analyses of input and output; due to limited power in our sample, we are unable to consider all potential contributing variables (e.g., the same dataset was analyzed in a previous study and found that mothers’ work schedules were associated with vocabulary knowledge at 17 months (Laing & Bergelson, 2019); we do not analyze that variable further here due to our limited sample size, though we note that in a preliminary analysis number of siblings and maternal work schedule were unrelated; see Supporting Information S1: S4). Our goal here is to make motivated decisions that we clearly describe, provide some alternative analytic choices in the supplementals, and to share the data with readers such that they are free to evaluate alternative approaches.

### 2.1 | Participants

The present study was conducted according to guidelines laid down in the Declaration of Helsinki, with informed consent obtained from a parent or guardian for each child before any assessment or data collection. All procedures involving human subjects in this study were approved by the Institutional Review Board at the University of Rochester. Forty-four families in New York State completed the year-long study. Infants (21 females) were from largely middle-class households; 33 mothers had attained a B.A. degree or higher. Based on parental report, no infants had speech- or hearing-relevant diagnoses; none were low birth weight (all >2500 g); 42 were white, two were from multi-racial backgrounds. All infants heard >75% English on a regular basis and lived in two-parent homes. Two participants were dizygotic twins; we retain one twin in the current sample, considering the other only as a sibling.^3^ Thus our final sample size was 43 infants.

#### 2.1.1 | Sibling details

Sibling number was computed based on parental report in the demographics questionnaires completed at 0;6 (Sibling number range: 0–4). Siblings were on average 4.11 years older than the infants in this study (SD: 4.01 years, R: 0–17 years).^4^ All siblings lived in the household with the infant full time, apart from one infant who had two older half siblings (and no other full siblings) who lived with their other parent part of the time. Both older siblings were present for at least some of the monthly recordings. One family had a foster child live in the home for 2 months of data collection, who is not accounted for in our data; the target infant had one sibling. All siblings were older than or of the same age as the infant in question.

### 2.2 | Materials

#### 2.2.1 | Parental report data

To index each child’s language abilities, we draw on data from vocabulary checklists (MacArthur-Bates Communicative Development Inventory, hereafter CDI, Fenson et al., 1994), administered monthly from 0;6 to 1;6, along with a demographics questionnaire; each month’s CDI survey came pre-populated with the previous month’s answers to save on reduplicated effort. Because the majority of infants did not produce their first word until around 0;11 according to CDI reports (*M* = 10.67 months, SD = 2.23), we use CDI data from 0;10 onwards in our analysis. CDI production data for each month is taken as a measure of the infants’ lexical development. CDI data for production has been well validated by prior work, including work in this sample (Frank et al., 2021; Moore et al., 2019). Of the intended 13 CDIs collected for each of the 43 infants, 26 were missing across 11 infants (leaving 559 CDIs in total). Four infants had four CDI data-points missing, while the majority (*n* = 5) had only one missing data-point.

#### 2.2.2 | Home-recorded video data

Every month between 0;6 and 1;5, infants were video-recorded for 1 h in their home, capturing a naturalistic representation of each infant’s day-to-day input. Corresponding to our CDI measures above, here we draw on recordings taken between 0;10 and 1;5. We did not ask families to ensure certain family members were or were not present; our video recordings capture whoever was home at the time families opted to schedule. Here we draw on data from the two caregivers who produced the most words in each recording; in 86% of cases this was the mother, and 10% of cases the father. Fathers produced the second highest number of words in 48% of cases (see Supporting Information S1: S3 for a full breakdown of speakers classed as caregivers in the dataset; note that the two main caregivers might differ for a given infant across sessions). At the child level, the modal caretaker across the eight videos was the mother for 37 infants, father for 4 infants, and grandmother for the remaining 1 infant. One infant had an equal number of sessions (four each) with the mother and babysitter as the most talkative caregiver. Infants wore a hat with two small Looxcie video cameras attached, one pointed slightly up, and one pointed slightly down; this captured the scene from the infants’ perspective. In the event that infants refused to wear the hats, caregivers wore the same kind of camera on a headband. Additionally, a camcorder on a tripod was set up in the room where infants and caretakers were interacting to capture a broader view; families were asked to move this camcorder if they changed rooms. The dataset includes eight videos for each child, one for each month that we analyzed.

Object words (i.e., concrete nouns) deemed to be said to, by, or loudly and clearly near the target child were annotated by trained coders for several properties of interest to the broader project on noun learning. Here we examine annotations for speaker, that is, who produced each noun, and object presence, that is, whether the noun’s referent was present and attended to by the infant (see “Derived Input Measures” below).

##### Derived input measures

Two input measures were derived based on the individual word level annotations of concrete nouns directed to or near the target child in this corpus, each pertaining to an aspect of the input that is established as important in early language learning: **overall household input** (how many concrete nouns does each infant hear? Note that this measure only includes speech produced directly to or close by the target child; see example below) and **object presence** (what proportion of this input is referentially transparent?), detailed below. The original dataset coded for synthesized speech from toys/electronics and speech from speakers on screens or radio; these were excluded here, alongside speech from experimenters (from equipment setup/takedown; 4714 tokens excluded from the video data taken between 10 and 17 months), leaving 69,741 tokens in the input analysis in total.

Neither of household input or object presence are, in our view, interpretable as “pure” quality or quantity input measures; we hold that quality and quantity are inextricably linked in general, and specifically we include (by design) only object words that the recordings suggest were possible learning instances for the infants who heard them, wherein quantity and quality are conflated. This included only concrete, imageable nouns that were addressed directly to the child (e.g., “Have you got your toy *bear*?”), or sufficiently loud and proximal that they were clearly audible to the child (e.g., “Can you pass me the toy *bear*?,” directed at the sibling while mother, infant, and sibling play on the rug). As mentioned above, only speech produced in the infant’s immediate surroundings (i.e., speech that would have been clearly heard by the target infant) was coded.

*Household Input* reflects how many nouns infants heard in the recordings from their two main caregivers (operationalized as the two adults who produced the most nouns in each recording; see above) and (where relevant) siblings. Input from speakers (adults or children) other than these two caregivers (and siblings) was relatively rare during video recordings, accounting for 0.61% of input overall (SD = 3.81%), and is excluded from our analysis. This measure of the early language environment is based on evidence showing strong links between the amount of speech heard in the early input and later vocabulary size (Anderson et al., 2021). We specifically consider only nouns produced by speakers in the child’s environment, directed to or produced clearly near the child, as nouns are what was annotated in the broader SEEDLingS project from which these data are taken; concrete nouns are acquired earlier in development in English and cross-linguistically (Braginsky et al., 2019). As in any sample of naturalistic interaction, the number of nouns correlates highly with the number of words overall (e.g., based on automated analyses of adult word counts vs. manual noun-only annotations, Bulgarelli & Bergelson, 2020). Thus, noun count in the monthly hour of video data serves as our household input proxy.

*Object Presence* was coded as “yes,” “no,” or “unsure” for each object word annotated in the home recordings, as produced by the two main caregivers detailed above, based on trained annotators’ assessment of whether the referent of the word (i.e., the object) was present and attended to or touched by the child or the caregiver. For example, if the caregiver was pointing at a ball while the saying the word *ball*, this was coded as “yes.” If the infant was holding (but not looking at) a bottle while the caregiver said *bottle*, this would also be coded as “yes.” On the other hand, if the caregiver refers to shoes that need to get put away in the other room, that would be coded as “no,” as it was not present during object labeling. In the video data, 145 instances (0.28% on average per infant) of object presence were marked as unsure; these instances were not included in this analysis.

### 2.3 | Data analysis

While we set out to test the hypotheses outlined above, aspects of our analysis were exploratory in nature. In respect of this, and on the advice of a helpful anonymous reviewer, we focus on descriptive and confirmatory measures of analysis through data visualization and effect size reporting alongside significance testing. For each key variable tested, we present these three avenues for understanding the data, alongside any further follow-up exploratory analyses, where appropriate.

All reported models were generated in R (R Core Team, 2019) using the *lmerTest* package to run linear mixed-effects regression models when needed (Kuznetsova et al., 2017). *p*-values were generated by likelihood ratio tests resulting from nested model comparison. All models include infant as a random effect. Since the raw data were highly skewed, log-transformed data and/or proportions were used for the reported models and model comparisons (1 was added to the raw infant production data of all infants before log-transformation to retain infants with vocabularies of 0); this brought our data closer to normality, though note that the model comparisons run here are not overly sensitive to skewed data. That said, given that all of our variables of interest (CDI score, household input and object presence) did differ significantly from normality by Shapiro tests, we opted to run non-parametric tests (two-sample two-tailed Wilcoxon Tests) on non-transformed data for all post hoc comparisons, where divergences from normality are more likely to have an outsize effect. Where multiple post hoc comparisons are run on the same dataset, Bonferroni corrections are applied (e.g., with an adjusted *p*-value threshold of .025 for 2 between-group comparisons). Unless otherwise specified, all figures display non-transformed data for interpretive ease.

While we have a substantial amount of data for each participant, our limited *n* means we are under-powered to consider multiple demographic variables simultaneously given the data distribution (e.g., sibling number and sex, see Table 1; as luck would have it both infants with three siblings were girls and both with four were boys). There were no correlations between sibling number or child word production and maternal age/education/work hours. See Supporting Information S1: S4 for further details.

## 3 | RESULTS

Our analyses consider infants’ total productive vocabulary^5^ alongside our two input measures—nouns in household input and extent of object presence in the input—as a function of sibling number.

**FIGURE 1:**
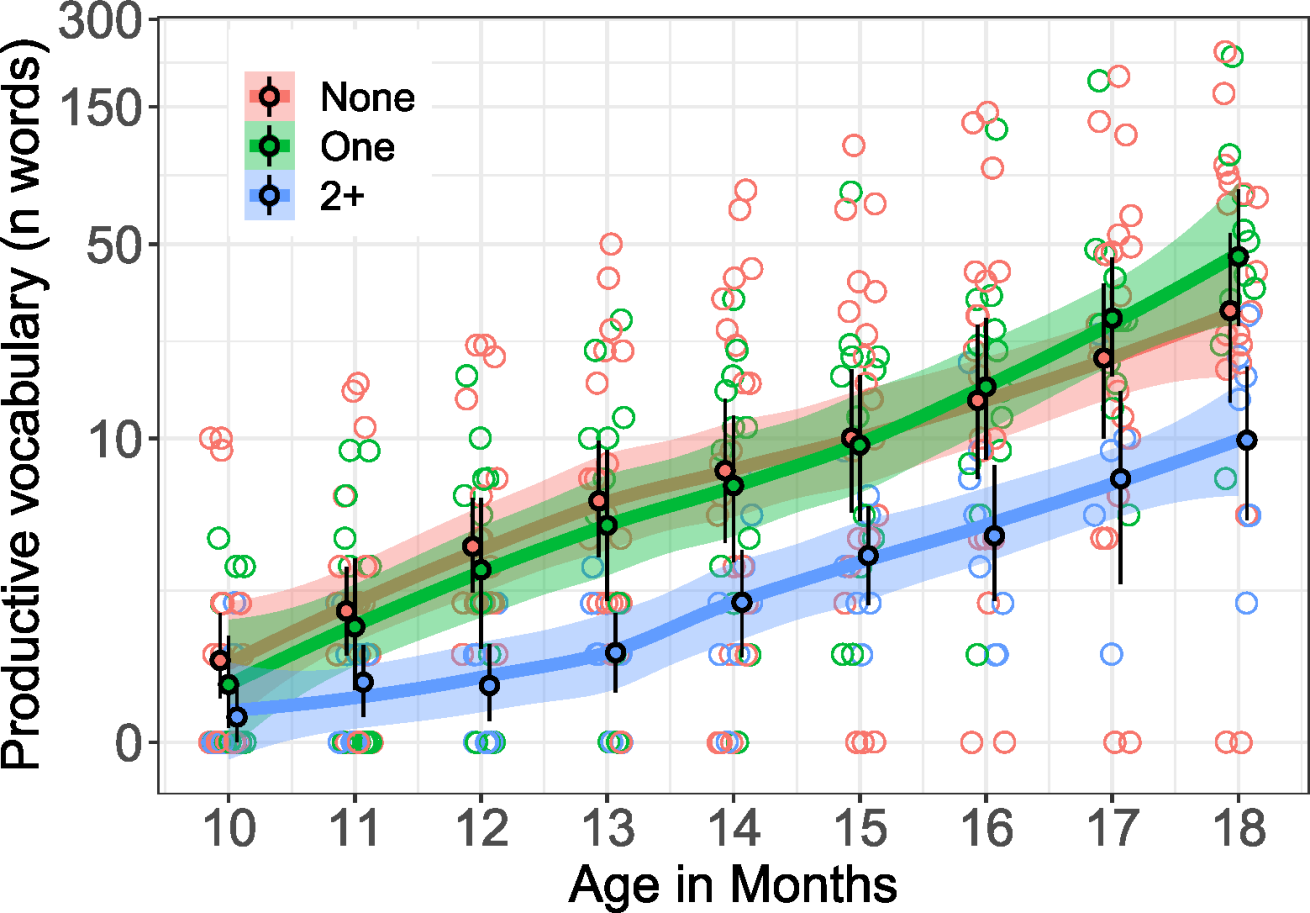
Reported productive vocabulary acquisition (CDI) over time (*n* = 42; one child was an outlier, and was removed from the CDI analysis and this figure; see text for details). Colors denote sibling group; line with colored confidence band reflects local estimator (loess) fit over individual infants’ vocabulary at each month. Filled circles indicate mean with bootstrapped CIs computed over each month’s data. Open circles (jittered horizontally) show individual infants’ vocabulary size at each month. Y-axis utilizes log-transformed vertical spacing for visual clarity.

Vocabulary development was highly variable across the 43 infants, according to the CDI data we had available. By 18 months, two infants produced no words (taken from 36 available CDIs at this time-point), while mean productive vocabulary size was 60.28 words (SD = 78.31, Mdn = 30.50). Three infants had substantially larger-than-average (3SDs above the monthly mean) vocabularies at certain time-points in the data; we counted one of these infants as an outlier and remove this child’s data from the CDI analysis given that their vocabulary was higher for multiple consecutive months (1;1–1;6). The other two infants had higher vocabularies at 10–11 months only (when variance was quite limited, see Figure 1), and were retained to maximize data inclusion. This left 42 infants (19 females) in the analysis of vocabulary size. Infants had one sibling on average (*M* = 0.86, Mdn = 1, SD = 1.10). See Table 1.

### 3.1 | Effect of siblings on infants’ productive vocabulary

We first modeled the effect of siblings on reported productive vocabulary. We explored three possible variations on how to represent the sibling effect: a binary variable (0 vs. >0 siblings), aggregated groups (None vs. One vs. 2+ siblings), and discrete sibling number (0–4 siblings), comparing the following nested model structures, where (1) is the baseline model and (2) includes siblings as the variable of interest.

1. Vocabulary size (log-transformed) ~ age (months) + (1|subject)
2. Vocabulary size (log-transformed) ~ siblings [binary, group or discrete] + age (months) + (1|subject)

In our sample, simply having siblings (i.e., as a binary variable) did not predict CDI productive vocabulary size, while both discrete sibling number and sibling group did. See Table 2.^6^

Having more siblings was associated with a smaller vocabulary size over the course of early development. This is consistent with previous findings (Hoff-Ginsberg, 1998; Pine, 1995). We find that for each additional sibling, infants were reported to have produced 24% fewer words on average.^7^ The “sibling effect” is thus present in our data.

In terms of our grouped sibling variable (i.e., 0 vs. 1 vs. 2+ siblings), infants with one sibling produced 64 words on average at 18 months, which is, on average, five words more than their firstborn peers. Consistent with the model results shown in Table 3, infants with two or *more* siblings produced substantially fewer words at 18 months than those with one or no siblings (based on the raw data: None vs. 2+: 59 vs. 13; One vs. 2+: 64 vs. 13). Post hoc Wilcoxon Rank Sum tests comparing reported productive vocabulary at 18 months (where there’s the widest vocabulary range) revealed significantly larger vocabularies for infants with one sibling compared to those with two or more siblings (*W* = 5, *p* = .004, CI = [−72.00, −12.00]), but no difference between infants with one sibling and those with no siblings (*W* = 79.50, *p* = .631, CI = [−34.00, 34.00]). See Table 4.

### 3.2 | Effect of siblings on infants’ input

Having established that infants’ productive vocabulary varied as a function of sibling number in all but the binary version of the measure (0 vs. >0 siblings), we turn to our input measures to test whether *input* varied by a child’s sibling status. For these analyses we report here the group sibling division (0 vs. 1 vs. 2+) as this lets us keep relatively similar *N*s across groups, thus making variance more comparable for post-hoc comparisons (the discrete sibling number (0–4) version is reported for completeness in Supporting Information S1: S5; results hold for both input variables). We also now include the child who was a multi-month vocabulary outlier above, given that input and vocabulary are not tested in the same model. One infant of the full sample of 43 infants was an outlier in that they heard substantially more input words and words with object presence than all the other infants in the sample in four of their recording sessions. Given that these sessions were not contiguous, we opted to keep this infant in the analyses reported below, though all results hold when they are removed from our sample (see Supporting Information S1: S6).

While we didn’t have strong a priori expectations about how overall input or object presence would vary by age or sex, these were included in initial model comparisons to see if they improved fit alongside a random effect of infant. Both variables improved fit for the input model, and only age did for the object presence model. Thus our baseline models include these sets of control variables, respectively. See Table 5 for final model estimates.

#### 3.2.1 | Caregiver input

We tested overall quantity of input (aggregated across the two main caregivers in each session, as outlined above, and siblings) in our model alongside age, sex, and subject, as noted above, and a significant effect was found for the effect of sibling group (*χ* ^2^(2) = 9.09, *p* = .011, *R* ^2^ = 0.59). Averaging across infants, those with one sibling heard on average 8% fewer words than those with no siblings in any given hour-long recording, while infants with two or more siblings heard 45% fewer words than those with no siblings.

We then ran post hoc tests to compare mean amount of input across sibling groups; these showed a significant difference in average input received between infants with one sibling versus those with two or more siblings (*W* = 11, *p* = .002, CI = [−120.87, −39.87]; Bonferroni-corrected *p*-threshold = .025 for all reported Wilcoxon tests) while amount of input did not differ between infants with no siblings and those with one sibling (*W* = 147, *p* = .723, CI = [−40.50, 57.50]).

**FIGURE 2:**
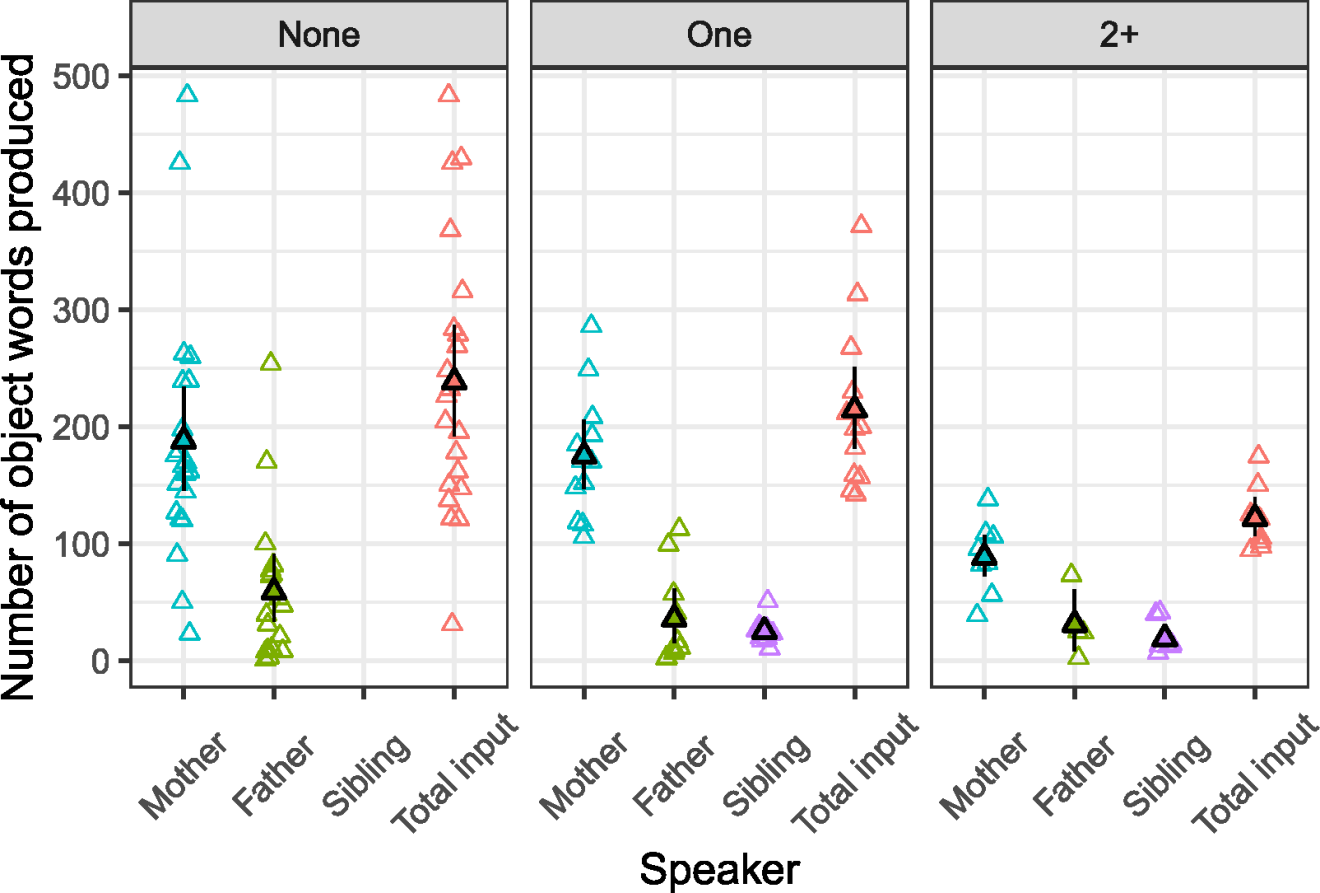
Mean number of words produced by mothers, fathers, and siblings, as well as total family input (mean input from mother + father + sibling(s)), across sessions recorded between 10 and 17 months. Open triangles represent values for individual infants; filled triangles show group means. In the case where the infant had two mothers, mean maternal input is shown. See Supporting Information S1: S7 for a month-by-month visualization of caregiver input.

While we operationalized caregiver input in our models as input speech from the two adults who produced the most words in any given session, in 87% of cases this was the mother or father. Considering mothers and fathers specifically, maternal input accounted for 75% of object words in the data overall (*M* = 163.14 words, Mdn = 151.69, SD = 90.73).^8^ Fathers accounted for an average of 18% (*M* = 58.42, Mdn = 32.50, SD = 64.88), while infants with siblings received around 12% of their input from their brothers and sisters (*M* = 22.97, Mdn = 18, SD = 18.49). See Figure 2, showing the raw values of input from mothers, fathers, and siblings, which are consistent with the group trends reported above. As well as the overall input being greater for firstborns compared with infants with one or 2+ siblings, note also that the variance is greater for this group, and decreases as sibling number increases. This is shown in the SDs reported in Table 4, and in the data points visualized in Figure 2.

Overall, for infants who had siblings, at least one other child was present in 76% of video recordings (*n* = 133 recordings, SD = 24%). Wilcoxon Rank Sum tests comparing mean monthly input showed no difference between the amount of sibling input received by infants with one sibling compared with those with two or more siblings (*W* = 31, *p* = .071, CI = [−14.50, 2]). Looking at mothers and fathers individually, infants with two or more siblings heard significantly less input from their mothers than those with one sibling (*W* = 5, *p* < .001, CI = [−124.88, −41.92]), while there was no difference between those with one versus no siblings (*W* = 125, *p* = .985, CI = [−48.23, 51.50]). Finally, amount of paternal input did not differ between groups (one vs. none: *W* = 108, *p* = .388, CI = [−12.37, 57.62]; one vs. 2+: *W* = 21, *p* = .945, CI = [−74.33, 56.54]).

#### 3.2.2 | Object presence

On average, 60% of annotated utterances included a referent that was present and attended to by the infant (Mdn = 0.61, SD = 0.12). See Table 4. Consistent with our hypothesis that infants with more siblings would hear fewer words in referentially transparent conditions (i.e., they would experience lower object presence) than those with fewer siblings, our models reveal a significant effect for sibling group on object presence (*χ* ^2^(2) = 27.52, *p* < .001, *R* ^2^ = 0.55).

**FIGURE 3:**
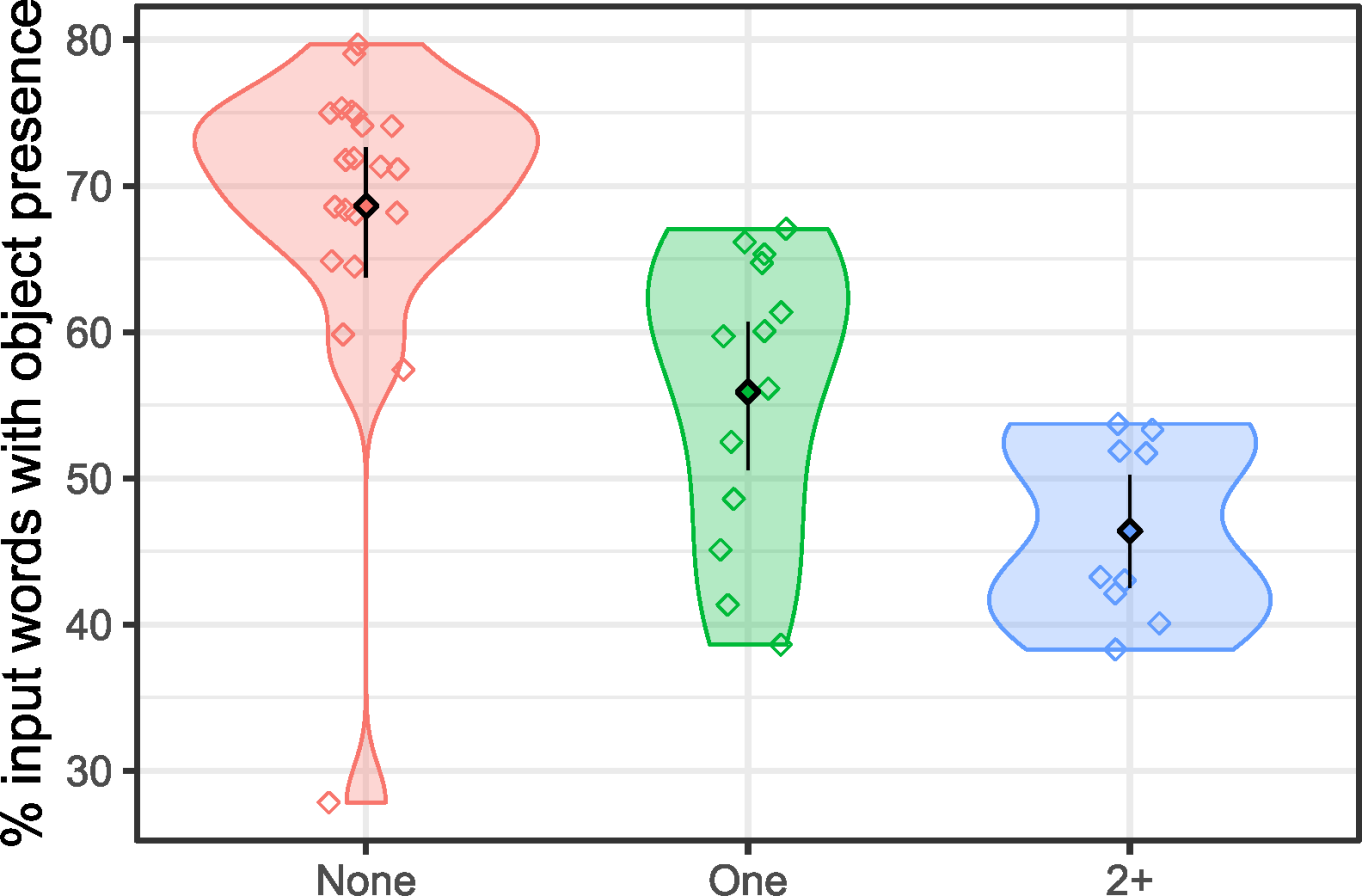
Percentage of input words produced with object presence across sibling groups. Error bars and filled diamonds show 95% CIs and mean proportion of object presence across sibling groups. Open shapes indicate mean proportion of object presence per infant, collapsing across age and jittered horizontally for visual clarity. See Supporting Information S1: S7 for a month-by-month visualization of object presence.

Descriptively, infants with no siblings experienced on average 32% more object presence in their input than those with two or more siblings, and 19% more than those with one sibling. Post hoc comparisons revealed significant between-group differences: infants with no siblings experienced significantly more object presence than those with one sibling (*W* = 240, *p* < .001, CI = [0.07, 0.20]; Bonferroni-corrected *p*-threshold = 0.025). Likewise, infants with one sibling experienced significantly more object presence those with two or more siblings (*W* = 25, *p* = .025, CI = [−0.18, −0.01]). See Table 5 and Figure 3.

#### 3.2.3 | Sibling presence

So far, our analysis takes into account the differences in input based on whether or not the target child has a sibling, but does not directly consider whether sibling presence in the recordings affected these variables. That is, if it is the active presence of the sibling that affects how the caretaker interacts with the target child, then we would expect to see a difference in our input measures when the sibling is present versus absent. On the other hand, if the very fact of having a sibling changes the way that a caregiver interacts with the infant regardless of whether any sibling is actually present on the scene, then no difference should be observed. While sibling presence in each recording was not coded directly in the dataset, for this exploratory analysis we can get at this with an admittedly imperfect proxy: did the sibling produce nouns in the recording? If yes, we can safely assume they are present; if not we (less safely, but reasonably for initial exploratory purposes) assume they are not. As reported above, by this measure, at least one sibling was present in 76% of recordings for the infants who had a sibling.

**FIGURE 4:**
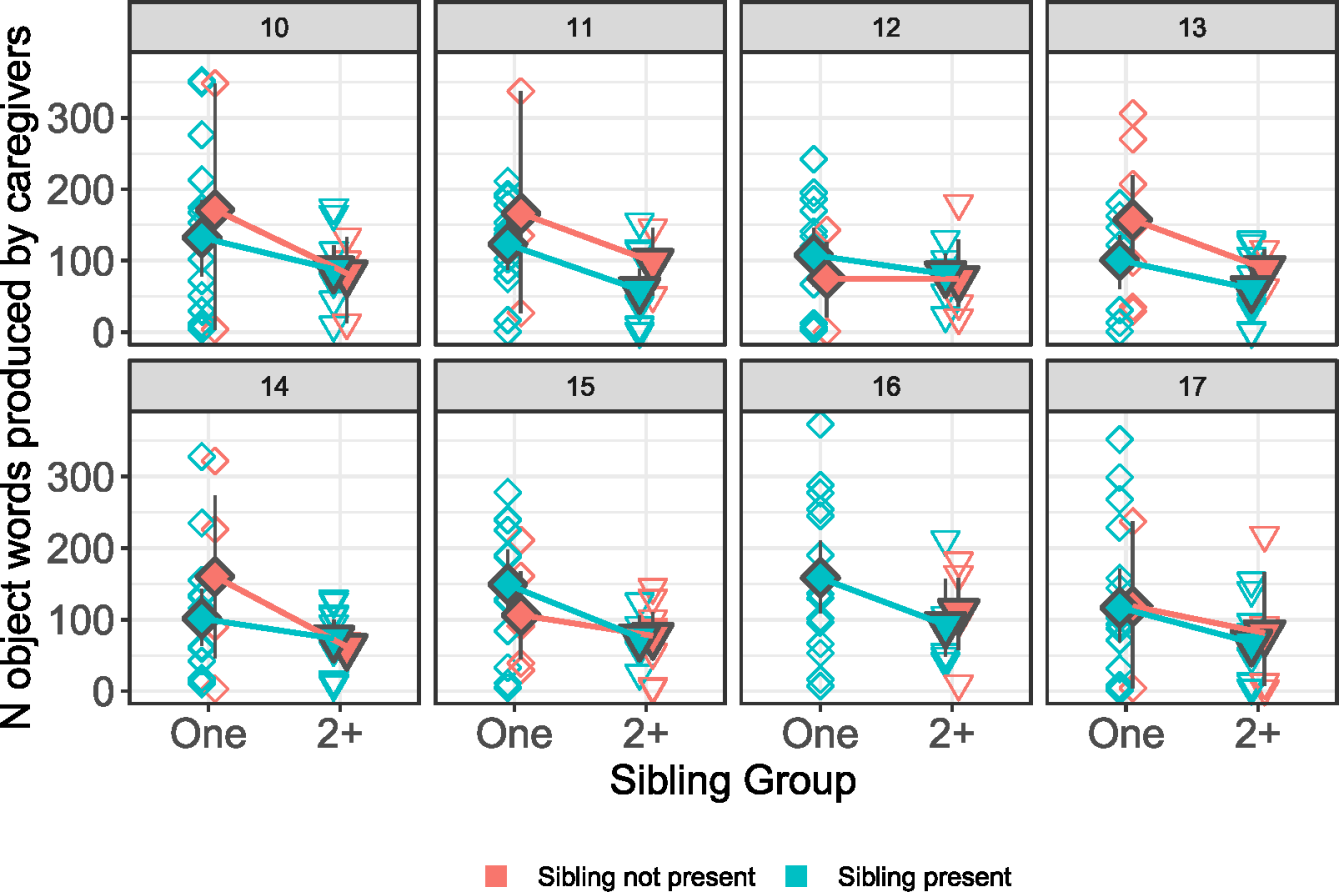
Difference in number of input words in infants’ input according to whether or not a sibling or siblings were present during the time of recording, for each month of data. Infants with no siblings were not included in the plots for visual ease. Open diamonds represent individual infants in the data; filled diamonds represent means and 95% CIs; colors represent presence or absence of siblings during the recording session.

**FIGURE 5:**
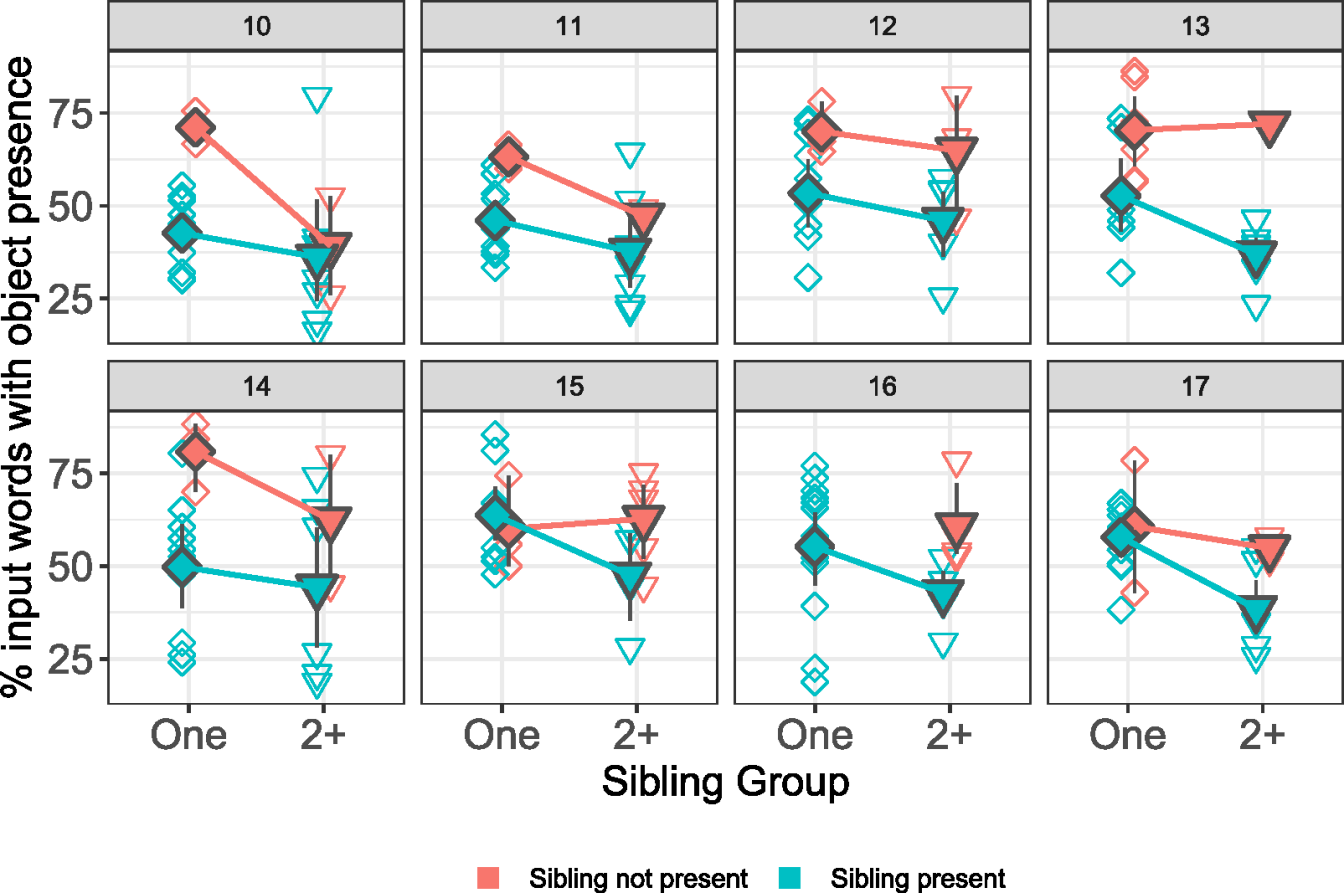
Difference in % of object presence in infants’ input according to whether or not a sibling or siblings were present during the time of recording, for each month of data. Infants with no siblings were not included in the plots for visual ease. Open shapes represent individual infants in the data; filled shapes represent means and 95% CIs; colors represent presence or absence of siblings during the recording session.

Since the presence of a sibling in any given infant’s data changed month-on-month (i.e., sometimes the sibling was present and sometimes they were not), and since our measure of sibling presence is imperfect, we opt here to describe the pattern of data without drawing any strong conclusions from statistical models. Descriptively, the presence of a sibling affected the amount of object presence in the data, but not the amount of input. See Table 6 and Figures 4 and 5. Overall, the presence of a sibling negatively affected object presence, and this was consistent over time; when a sibling was present, infants in both groups heard less object presence. This effect was stronger for infants with two or more siblings (though note that it is unclear from our measure how many siblings were present, and it is possible that only one sibling was present in the recording), and overall this was true regardless of whether the sibling was present, or whether the infant was alone with the caregiver. The picture is less clear for caregiver input, where the presence of a sibling has a more variable effect on the number of object words produced by caregivers (see Figure 4), particularly for infants with only one sibling. This supports our findings above, suggesting that the presence of one additional child does not have any negative effects on the amount of input that caregiver provide. However, input was consistently lower for the group with two or more siblings, and this was true regardless of whether or not a sibling was present; again, this is consistent with the findings reported above for this input measure.

## 4 | DISCUSSION

We investigated the nature of infant language development in relation to number of children in the household. Previous research found a delay in lexical acquisition for later-born infants (Fenson et al., 1994; Hoff, 2006), with differences in input across birth order reported as a root cause. Our results add several new dimensions to this, by testing for differences across more versus fewer older siblings, and by looking at input during child-centered home recordings. Infants with more siblings were reported to say fewer words by 18 months, heard fewer nouns from their parents, and were less likely to be attending to an object when hearing its label.

Importantly, and in contrast with some previous research (Hoff-Ginsberg, 1998; Oshima-Takane & Robbins, 2003), infants with one sibling showed no decrement in lexical production and minimal reduction in input in comparison to first-born infants. That is, our results suggest that simply having a sibling does not contribute to input or vocabulary differences across children (as measured here), while having more than one sibling seems to do so. Indeed, infants with zero and one sibling had similar results for productive vocabulary, and parental noun input overall (though not object presence). Moreover, parental input was not affected by the presence or absence of the sibling in the room. In contrast, infants with two or more siblings said fewer words, and also heard fewer input words overall.

With regards to object presence, having siblings made it less likely to hear an object label when attending to it, and this effect increased with sibship size (i.e., children with more siblings heard input containing a lower proportion of object presence). Unlike for total parental noun input (which was reduced for 2+ siblings but not modulated based on whether siblings were present in the recordings), reduced object presence for children with more siblings was particularly notable in recordings with siblings present.

### 4.1 | The sibling effect

When we considered the effect of sibling status—that is, whether or not infants had any siblings, disregarding specific sibling number—our findings showed that having siblings made no difference to infants’ lexical production capacities. This contrasts with Hoff-Ginsberg (1998), who found that, by 18 months, laterborns exhibit lower language skills. However, Oshima-Takane et al. (1996) found no overall differences between first- and second-born children across a range of language measures taken at 21 months. Our results suggest that considering *sibling quantity* may be a more sensitive way to reveal demographic effects than their (coarser-grained) first-versus later-born status. We find that the more older siblings a child had, the lower their reported productive vocabulary at 18 months. This adds to findings from Fenson et al. (1994), who found a weak but significant negative correlation between birth order and production of both words and gestures. Controlling for age, our model showed that infants with 2 or more siblings produced 46 fewer words than the average 59 words produced by firstborns in our data by 18 months.

While infants with more siblings heard less input speech overall, having one sibling did not significantly reduce the number of nouns in an infant’s input. This is in direct contrast with reports from the literature; Hoff (2006) states that “when a sibling is present, each child receives less speech directed solely at…her *because mothers produce the same amount of speech whether interacting with one or two children*” (p. 67, italics added). While this does not appear to be the case in the present dataset, it may be due to the circumstances of the home-recorded data: while siblings were present in many of the recordings (76% of recordings in which the target child had a sibling), given the focus of the data collection, parents may have had a tendency to direct their attention—and consequently their linguistic input—more toward the target child; our samples also differed in other ways (e.g., sociocultural context) that may have influenced the results as well. Alternatively, our results may diverge from those of Hoff (2006) due to the nature of our input measure, which only took nouns into account. That said, we find this alternative explanation unlikely given work by Bulgarelli and Bergelson (2020) showing that nouns are a reliable proxy for overall input in this dataset, suggesting that this measure provides an appropriate representation of overall input directed at the target child.

In contrast to the other results, our analysis of object presence showed a more linear “sibling effect.” In this case, even having one sibling led to fewer word–object pairs presented in the input. This was true regardless of whether or not other siblings were present, but object presence was further negatively affected by the presence of a sibling in the room. Presence of a labeled object with congruent input speech has been found to support early word learning across several studies. For instance, Bergelson and Aslin (2017) combined analysis of this home-recorded data at 6 months with an experimental study to show that word-object presence in naturalistic caregiver input correlated with comprehension of nouns (tested using eye-tracking). Relatedly, Gogate et al. (2000) propose that contingent word production supports the learning of novel word–object combinations, with “multimodal motherese”—whereby a target object word is produced in movement or touch-based synchrony with its referent—supporting word learning. More broadly, lower rates of referential transparency for common non-nouns like *hi* and *uh-oh* have been proposed to potentially explain why these words are learned later than common concrete nouns (Bergelson & Swingley, 2013). While the present results on object presence don’t speak directly to word learning, they do suggest that this potentially helpful learning support is less available for children with more siblings.

### 4.2 | Siblingese as a learning opportunity?

We also found that infants with siblings did not hear much speech from their older brothers and sisters. Similar findings are reported in a lab-based interaction study by Oshima-Takane and Robbins (2003), who found that older siblings rarely talked directly to the target child; instead, most input from siblings was overheard speech from sibling–mother interactions. One possibility raised by these results is that perhaps parents are able to compensate or provide relatively similar input and learning support for one or two children, but once children outnumber parents, this balancing act of attention, care, and time becomes unwieldy. While the current sample is relatively limited and homogeneous in the family structures and demographics it includes, future work could fruitfully investigate this possibility by considering whether (controlling for other potential contributors like socioeconomic status, Hoff-Ginsberg, 1998) the presence of more caregivers (whether parents, relatives, or other adults) helps foster language development.

Alternatively, second-borns might “even out” with children with no siblings due to a trade-off between direct attention from the caregiver and the possibility of more sophisticated social–communicative interactions. For these infants there is still ample opportunity to engage with the mother in one-to-one interactions, allowing a higher share of her attention than is available to third- or later-borns. Furthermore, triadic interactions can benefit the development of a number of linguistic and communication skills (Barton & Tomasello, 1991; Dunn & Shatz, 1989). Second-borns may also benefit from overheard speech in their input, supporting the acquisition of nouns and even more complex lexical categories (Floor & Akhtar, 2006; Oshima-Takane et al., 1996). For infants with one sibling, the benefits of observing/overhearing interactions between sibling and caregiver, as well as the possibility for partaking in such interactions, may outweigh the decrease in some aspects of the input (i.e., in our data, only observed in object presence). Having more than one sibling may throw this off-balance, such that the possibilities for both supportive one-to-one input *and* more sophisticated interactions are simultaneously diminished.

Importantly, the present results make no claims about eventual outcomes for these children: generally speaking, regardless of sibling number, all typically-developing infants reach full and fluent language use. Indeed, some research suggests that sibling effects, while they may be clear in early development, are not always sustained into childhood; for example, twins are known to experience a delay in language development into the third year, but are quick to catch up thereafter (Dales, 1969; Tomasello et al., 1986). This demonstrates the cognitive adaptability of early development, which brings about the acquisition of language across varying and allegedly “imperfect” learning environments. Infants’ capacity to develop linguistic skills from the resources that are available to them—whether that is infant-directed object labels or overheard abstract concepts—highlights the dynamic and adaptable nature of early cognitive development, and a system that is sufficiently robust to bring about the same outcome across populations.

### 4.3 | Limitations

Of course, the “success” of early language development is defined by how success is measured. Here we chose word production as our measure of linguistic capability; we did not consider other equally valid measures such as language comprehension or early social-interaction skills. Similarly, our input measures focused on nouns; other lexical classes may reveal different effects, though they are generally far sparser in production until toddlerhood. Our analysis of vocabulary relied on parental report data; this method could have biased our first-born sample toward more accurate or larger vocabulary reports owing to their parents having more time and attention to spend observing their vocabulary development (see Kartushina et al., 2022 for a discussion of this possibility in light of the COVID-19 pandemic, though note the present data were collected in 2014–2016). In the supplementary materials, we provide validation data for the CDI relative to children’s own productions by running correlation tests between reported (CDI) vocabulary and the number of word types produced by each infant in the audio and video data. In short, for the 0 and 1 sibling groups there is a strong and significant positive correlation between the CDI at 18 months and child word types; for 2+ siblings this correlation is weaker and does not reach significance, though this is likely due to the small *n* in this group relative to the others (due to missing CDIs at 18 months, this includes data from *n* = 18/21, 11/12, and 7/9 children with 0, 1, and 2+ siblings, respectively); see Supporting Information S1: S8.

There is also some imbalance in group sizes across our data; our sample was not pre-selected for sibling number, and so group sizes are unmatched across the analysis. Including a larger number of infants with 2+ siblings may have revealed a different pattern of results. We might also expect that age of older siblings would affect the nature of the early linguistic environment, given that larger age difference is found to be a predictor of lower vocabulary size in the current literature (Gurgand et al., 2022; Havron et al., 2022); our sample did not allow us to link sibling age to number of words produced by that sibling, but future work may wish to take this into account. Finally, more work across wider and larger populations is necessary to unpack the generalizability of the present results. Our sample is reflective of average household sizes in middle-class families across North America and Western Europe (Office for National Statistics, 2018; United States Census Bureau, 2010), but it is not unusual in some communities and parts of the world for households to include between three and six children on average (Institute for Family Studies & Wheatley Institution, 2019). Adding to this, it is also necessary to consider cross-cultural differences in the way children are addressed by their parents, other caretakers, and other children (Bunce et al., 2020; Casillas et al., 2019; Shneidman & Goldin-Meadow, 2012). For instance, Bunce et al. (2020) find relatively similar rates of target child directed speech across US, Canadian, Argentinian, UK, Papuan, and Mayan samples, some differences in who the input comes from, and large effects of number of talkers present. These results suggest that caution is advisable before generalizing the current results to any other socio-cultural contexts, but also pose exciting open questions regarding what variability in experiences do—or don’t—change about early language interaction and development.

### 4.4 | Conclusion

Our results with English-learning infants in the US support prior findings from the literature showing that later-born infants have slower lexical acquisition than their first-born peers. However, we highlight an important difference from previous findings, namely that in the present sample, second-born infants show no such effect, while infants with more than two siblings have significantly smaller productive vocabularies at age 18 months. We also identified similar group differences in overall noun input and object presence. While we did not test these corresponding vocabulary and input measures directly, our results suggest that having more siblings affects a child’s early language environment, which in turn may lead to slower vocabulary growth in the first 18 months of life. We look forward to future studies considering the granularity of more versus fewer siblings, and how this relates to language abilities over the course of development.

## Supplementary Material

SI for Laing & Bergelson 2024

## Figures and Tables

**TABLE 1:** Sibling number by female and male infants (*n* = 42).

*n* Siblings	Female	Male	Total
0	9	12	21
1	6	6	12
2	2	3	5
3	2	0	2
4	0	2	2
Total	19	23	42

*Note*: One child was an outlier, and was removed from the CDI analysis and this table; see text for details.

**TABLE 2:** Output from likelihood ratio tests comparing regression models that predict the effects of sibling number (binary, grouped, and discrete variables) on vocabulary size.

Model	*df*	Chi square	*p* value	*R* ^2^
0 versus >0 siblings	1	2.13	.14	0.81
Sibling group	2	8.00	.02	0.81
Sibling number	1	6.08	.01	0.81

*Note*: Month was included in each model as a fixed effect; subject was included as a random effect. *R*^2^ values are included to reflect model goodness-of-fit, though we note that utility and interpretability of this metric for this model type is debated (see https://bbolker.github.io/mixedmodels-misc/glmmFAQ.html).

**TABLE 3:** Full model output from linear mixed effects regression models comparing language development over time in relation to sibling group.

Effect	Estimate	Std.error	*df*	*t* value	*p*
Intercept	−2.69	0.26	156.59	−10.27	<.001
SibGroupOne	−0.01	0.30	42.08	−0.05	.963
SibGroup2+	−0.94	0.33	42.84	−2.81	.007
Month	0.34	0.01	315.13	25.19	<.001

*Note*: Age in months was included as a fixed effect; subject was included as a random effect.

**TABLE 4:** Data summary of our two input measures and reported vocabulary size at 18 months.

	No siblings		1 Sibling		2+ Siblings	
			
Variable	Mean	SD	Mean	SD	Mean	SD
Productive vocabulary 18 months (CDI)	58.89	60.76	64.10	61.97	13.00	9.49
*N* input utterances, 10–17 months	213.33	101.51	196.27	53.96	117.36	26.46
% Object presence in input, 10–17 months	68.61	10.93	55.90	9.79	46.38	6.17

*Note*: Input measures represent input from the two adults who produced the most words in any given session, plus siblings.

**TABLE 5:** Full model output from linear mixed effects regression models comparing our two input measures (object words produced in caregiver input and object presence) over time in relation to sibling group.

Variable	Effect	Estimate	Std.error	*df*	*t* value	*p* value
Caregiver input	Intercept	4.88	0.18	185.99	27.46	<.001
	SibGroupOne	0.01	0.15	43.00	0.05	.960
	SibGroup2+	−0.49	0.17	43.00	−2.98	.005
	Month	0.03	0.01	301.00	2.95	.003
	SexM	−0.18	0.13	43.00	−1.41	.164
Object presence	Intercept	0.57	0.04	321.44	12.73	<.001
	SibGroupOne	−0.13	0.03	43.00	−3.81	<.001
	SibGroup2+	−0.22	0.04	43.00	−5.90	<.001
	Month	0.01	0.00	301.00	2.93	.004

*Note*: Age in months was included as a fixed effect in both models, sex was included in the caregiver input model only; subject was included as a random effect.

**TABLE 6:** Data summary of our two input measures according to presence or absence of siblings during the recording.

		1 Sibling		2+ Siblings	
		
Variable	Sibling presence	Mean	SD	Mean	SD
*N* input utterances, 10–17 months	Sibling not present	139.36	73.23	88.67	32.39
	Sibling present	126.92	38.69	75.01	26.29
% Object presence in input, 10–17 months	Sibling not present	70.26	14.39	64.60	12.10
	Sibling present	53.91	8.42	39.25	8.99

*Note*: Input measures represent input from the two adults who produced the most words in any given session, plus siblings.

## Footnotes

1 We’ve retained the term object presence for continuity with prior work using this variable but note that what this variable captures isn’t merely whether the object was present but rather whether it was present when the word for it was said aloud.

2 We also ran our input analysis using data sub-sampled from day-long audio recordings taken on a different day from the video data reported below; results were consistent with those outlined below for most analyses (see Supporting Information S1: S1).

3 Results were consistent when both twins were removed from the dataset, see Supporting Information S1: S2.

4 For six infants, siblings’ exact birthdates were not provided, and so age difference was estimated by subtracting the infant’s age at the time of data collection (6 months) from the sibling’s age in years, as listed on the questionnaire (e.g., if a sibling was 5 years old, they were classed as being 4.5 years older than the infant).

5 While in principle we could have just used noun productive vocabulary, in practice noun and total vocabulary is correlated >0.95 in this age range; we opted to retain the overall total vocabulary, as lexical class is not a straightforward notion in the early lexicon.

6 While our sample size and distribution leaves it statistically questionable to consider both sex and sibling number, for completeness we did also run a model that included sex in addition to age and sibling number (our primary variable of interest). Sex did not improve model fit over and above the effect of siblings in any of the three comparisons (*p*s all >.54).

7 Average difference in vocabulary size between 0 versus 1, 1 versus 2, 2 versus 3, and 3 versus 4 siblings at 18 months, as a percentage of firstborn vocabulary size.

8 One family in our sample had two mothers; rather than artificially assigning one parent to another category, we averaged both mothers’ input for this child; we acknowledge that this is an imperfect solution but found it better than the alternatives.
