# Supplementary material for: Analyzing the effect of sibling number on input and output in the first 18 months
Source: Infancy. Author manuscript; Available in PMC 2025 Mar 1. (PMC10900282; doi:10.1111/infa.12578)
Supplement: SI for Laing & Bergelson 2024 [file NIHMS1966132-supplement-SI_for_Laing___Bergelson_2024.pdf]

Analysing the effect of sibling number on input and output in the first 18 months: Supplementals

Catherine Laing[1] & Elika Bergelson[2]

[1] University of York, York, UK

[2] Harvard University, Cambridge, MA, USA

Author Note

Correspondence concerning this article should be addressed to Catherine Laing, Department of Language and Linguistic Science, University of York, Heslington, York, UK, YO10 5DD. E-mail: catherine.laing@york.ac.uk

Analysing the effect of sibling number on input and output in the first 18 months: Supplementals

The supplementary materials below present further analyses on our sample. We include this data here for sake of completeness and transparency, but do not include it in the reported results owing to small sample sizes and consistency across findings. All scripts and data for our analysis can be found on the project's OSF wiki at https://osf.io/rm3ec/.

The analyses below (S1:S8) show:

- S1: re-analysis of caregiver input variables using data from day-long audio recordings
- S2: re-analysis of caregiver input variables with both dizygotic twins excluded
- S3: summary of speakers classed as two main caregivers in our input data
- S4: correlations of input and output measures with maternal factors (age, education, work status) and number of siblings
- S5: re-analysis of caregiver input variables using discrete sibling number as our main variable of interest
- S6: re-analysis of caregiver input variables with one outlier infant
- S7: change to input variables over time
- S8: validating CDI scores with infant productions in the home-recorded data

Consistent with our main analysis, all analyses here draw on data from age 10 months, given that the majority of infants did not produce their first word until around 0;11, according to CDI reports. In addition, the twin removed from the main analyses is also removed from the data analysed here.

S1: Effect of siblings on infants' input - audio recordings

Our main results analyse input data from the hour-long video recordings taken in the home on a monthly basis. We opted to use the video data in the main analysis due to

consistency in data collection across time-points (one hour of video recording per month, per child). We re-ran our analysis using the home-recorded audio data, which captures a snapshot from a 16h daylong recording using a LENA device (https://www.lena.org/) worn in a vest. Three or four hours were transcribed per monthly recording; four hours in the 10-13-month recordings, and three hours in the 14-17-month recordings. These transcribed sections were selected based on the most talkative hours in each recording, as determined by an algorithm that ranked hour-long subregions according to LENA's CVC and CTC metrics over the entire 16h recording.

Initial model comparisons including the caregiver input/object presence as a dependent variable and age and sex as fixed effects showed no effect for sex on either measure, but an effect of age on both. Age, but not sex, was thus included in our regression models; data was included from age 10 months onwards.

We tested our two input variables using nested model comparisons, where (1) is the baseline model and (2) includes siblings as the variable of interest.

1. caregiver input/object presence (log-transformed) ~ age (months) + (1|subject)
2. caregiver input/object presence (log-transformed) ~ sibling group + age (months) + (1|subject)

Outputs from these model comparisons and full model outputs including estimates are shown in Tables 1 and 2. Results were consistent with the video data for object presence, where a significant effect was found for sibling group. Unlike the video data, this effect was not found in the model testing overall input.

Table 1

Results of nested model comparisons testing the effect of sibling group on caregiver input/object presence in the audio data. Linear mixed-effects regression models compared our two input measures (object words produced in caregiver input and object presence) in relation to sibling group (0 vs. 1 vs. 2+) in the audio data. Age in months was included as a fixed effect; subject was included as a random effect.

Model	Df	Chisq	p value	R^2
Caregiver input	2	2.75	0.252	0.69
Object presence	2	19.49	<0.001	0.44

Table 2

Full model outputs from two linear mixed effects regression models comparing our input measures (object words produced in caregiver input and object presence) in relation to sibling group (0 vs. 1 vs. 2+), for the audio data. Age in months was included as a fixed effect in both models and subject was included as a random effect.

Variable	Effect	Estimate	Std. Error	df	t value	p value
Caregiver input	Intercept	5.24	0.18	168.09	29.34	<0.001
	SibGroupOne	0.12	0.20	43.00	0.62	0.539
	SibGroup2+	-0.28	0.22	43.00	-1.27	0.212
	month	0.03	0.01	301.00	2.97	0.003
Object presence	Intercept	0.38	0.04	341.20	8.50	<0.001
	SibGroupOne	-0.09	0.03	43.00	-3.11	0.003
	SibGroup2+	-0.15	0.03	43.00	-4.69	<0.001
	month	0.02	0.00	301.00	6.46	<0.001

S2: re-analysis of caregiver input variables with both dizygotic twins excluded

As stated in the main paper, there was one pair of dizygotic twins in the original dataset. We retain one infant in the main analysis in order to maximise sample size, but we acknowledge that this is not a perfect solution given that the presence of another twin in the home (counted as a sibling in the main analysis) may affect caregiver interactions in a different way than the presence of an older sibling. By removing both twins from our data and re-analysing the input measures, we can confirm that our results were consistent with and without one of the twins included in our data (n=42). See Tables 3 and 4.

Table 3

Results of nested model comparisons testing the effect of sibling group on caregiver input/object presence in the video data, with both dizygotic twins removed from our sample (n=42). Linear mixed-effects regression models compared our two input measures (object words produced in caregiver input and object presence) in relation to sibling group. Age in months was included as a fixed effect for both models; subject was included as a random effect.

Model	Df	Chisq	p value	R^2
Caregiver input	2	8.41	0.015	0.58
Object presence	2	27.93	$<$0.001	0.55

Table 4

Full model output from linear mixed effects regression models comparing our two input measures (object words produced in caregiver input and object presence) over time in relation to sibling group, with both dizygotic twins removed from our sample (n=42). Age in months was included as a fixed effect in both models; subject was included as a random effect.

Variable	Effect	Estimate	Std. Error	df	t value	p value
Caregiver input	Intercept	4.77	0.16	252.93	28.95	<0.001
	SibGroupOne	-0.01	0.15	42.00	-0.09	0.930
	SibGroup2+	-0.49	0.17	42.00	-2.91	0.006
	month	0.03	0.01	294.00	2.95	0.003
Object presence	Intercept	0.56	0.05	316.86	12.44	<0.001
	SibGroupOne	-0.14	0.03	42.00	-3.98	<0.001
	SibGroup2+	-0.22	0.04	42.00	-5.92	<0.001
	month	0.01	0.00	294.00	3.05	0.003

S3: Speakers in the dataset

The table below provides a summary of which two caregivers produced the most words in each video recording analyzed in the main manuscript. This was usually the mother and/or the father. In the case where the infant had two mothers, their data was aggregated into the 'Mother' category; in four sessions from this family, the two mothers were the two main caregivers. There were 344 hour-long video recordings in total across the 43 infants in the data set; two speakers were present in 148 of these.

Table 5

Numer of sessions in which each adult speaker is one of the two main caregivers. There were 344 hour-long video sessions in total, and two caregivers were present in 146 of these.

Speaker	Caregiver 1	Caregiver 2
Mother	282	16
Father	45	83
Grandmother	11	24
Babysitter	4	4
Grandfather	1	5
Other Adult	1	7
Aunt	0	7
Uncle	0	2

S4: Correlations with maternal factors

We tested the extent to which maternal factors such as age, work status and education level correlate with number of siblings, productive vocabulary measures, and amount of input. Spearman's correlation tests confirmed a significant negative correlation between sibling number and words produced in the infant's input by the mother (rho=-0.36, p=.028). No other correlations were significant, though weak positive correlations were identified between amount of input and productive vocabulary, maternal age and sibling number, and maternal work hours and education. See Figure 1.

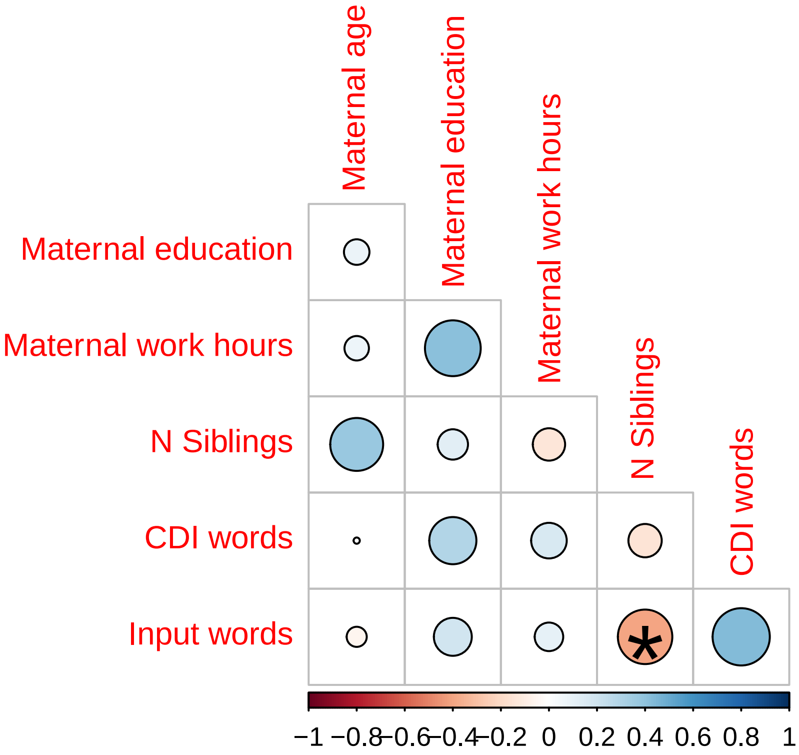

Figure 1. Correlation matrix showing Spearman's correlation coefficients between maternal factors (age, work status and education), number of siblings, and number of words reported to be produced by the child at age 18 months. Colours and circles represent direction and strength of correlation, whereby bolder colours and larger circles indicate a stronger correlation between variables; blue and red indicate positive/negative correlations, respectively. Asterisk indicates a significant p value at $p<.05$.

S5: Discrete sibling number

Our main analyses report the effects of sibling group (infants with 0, 1 or 2+ siblings) on our two input measures (number of input words produced by caregivers and object presence). We opted to go with this measure of sibling number owing to more balanced group sizes. Since discrete sibling number (raw number of siblings) also revealed siblings to be a significant predictor of vocabulary size at 18 months, here we re-run our input models using discrete sibling number as a fixed effect, instead of sibling group, to test whether this effect is consistent across our input measures.

Once again, initial model comparisons including the caregiver input/object presence as a dependent variable and age/sex as fixed effects showed no effect for sex on either measure, but an effect of age on both. Age, but not sex, was thus included in our regression models; data was included from age 10 months on-wards. As above, we tested our two input variables using nested model comparisons, where (1) is the baseline model and (2) includes siblings as the variable of interest.

1. caregiver input/object presence (log-transformed) ~ age (months) + (1|subject)
2. caregiver input/object presence (log-transformed) ~ sibling number + age (months) + (1|subject)

Outputs from model comparisons and full model outputs including estimates are shown in Tables 6 and 7. Results were consistent with those reported for sibling group, suggesting both characterizations of siblinghood capture similar relationships among our variables.

Table 6

Results of nested model comparisons testing the effect of discrete sibling number on caregiver input/object presence in the video data. Linear mixed-effects regression models compared our two input measures (object words produced in caregiver input and object presence) in relation to sibling number (R = 0-4). Age in months was included as a fixed effect; subject was included as a random effect.

Model	Df	Chisq	p value	R^2
Caregiver input	1	5.45	0.020	0.59
Object presence	2	30.24	<0.001	0.55

Table 7

Full model outputs from two linear mixed effects regression models comparing our input measures (object words produced in caregiver input and object presence) in relation to discrete sibling number (R = 0-4), for the video data. Age in months was included as a fixed effect in both models and subject was included as a random effect.

Variable	Effect	Estimate	Std. Error	df	t value	p value
Caregiver input	Intercept	4.81	0.16	274.49	30.17	<0.001
	n Siblings	-0.15	0.06	43.00	-2.41	0.020
	month	0.03	0.01	301.00	2.95	0.003
Object presence	Intercept	0.55	0.04	328.64	12.41	<0.001
	n Siblings	-0.08	0.01	43.00	-5.32	<0.001
	month	0.01	0.00	301.00	2.93	0.004

S6: Possible outlier for input data removed

One infant heard substantially more (>3SDs above the mean) nouns in their input and nouns with object presence than the other 42 infants in the main sample for four of their recording sessions. We retain this infant in the main analysis, but confirm here that results reported for sibling group were consistent when this child was removed from the analysis of input data (n=42). See Tables 8 and 9.

Table 8

Results of nested model comparisons testing the effect of sibling group on caregiver input/object presence in the video data, with one infant identified as an outlier removed from our sample (n=42). Linear mixed-effects regression models compared our two input measures (object words produced in caregiver input and object presence) in relation to sibling group. Age in months was included as a fixed effect; subject was included as a random effect.

Model	Df	Chisq	p value	R^2
Caregiver input	2	9.01	0.011	0.56
Object presence	2	26.06	<0.001	0.55

Table 9

Full model output from linear mixed effects regression models comparing our two input measures (object words produced in caregiver input and object presence) over time in relation to sibling group, with one infant removed who was identified as an outlier (n=42). Age in months was included as a fixed effect in both models, and subject was included as a random effect.

Variable	Effect	Estimate	Std. Error	df	t value	p value
Caregiver input	Intercept	4.73	0.16	262.71	28.89	<0.001

	SibGroupOne	0.08	0.14	42.00	0.54	0.593
	SibGroup2+	-0.44	0.16	42.00	-2.74	0.009
	month	0.03	0.01	294.00	2.84	0.005
Object presence	Intercept	0.56	0.05	312.10	12.26	<0.001
	SibGroupOne	-0.12	0.03	42.00	-3.67	<0.001
	SibGroup2+	-0.22	0.04	42.00	-5.74	<0.001
	month	0.01	0.00	294.00	3.02	0.003

S7: Change to input variables over time

We re-plotted Figures 2 and 3 from the main paper with age on the x-axis, to determine whether there was any notable age-related changes in these variables.

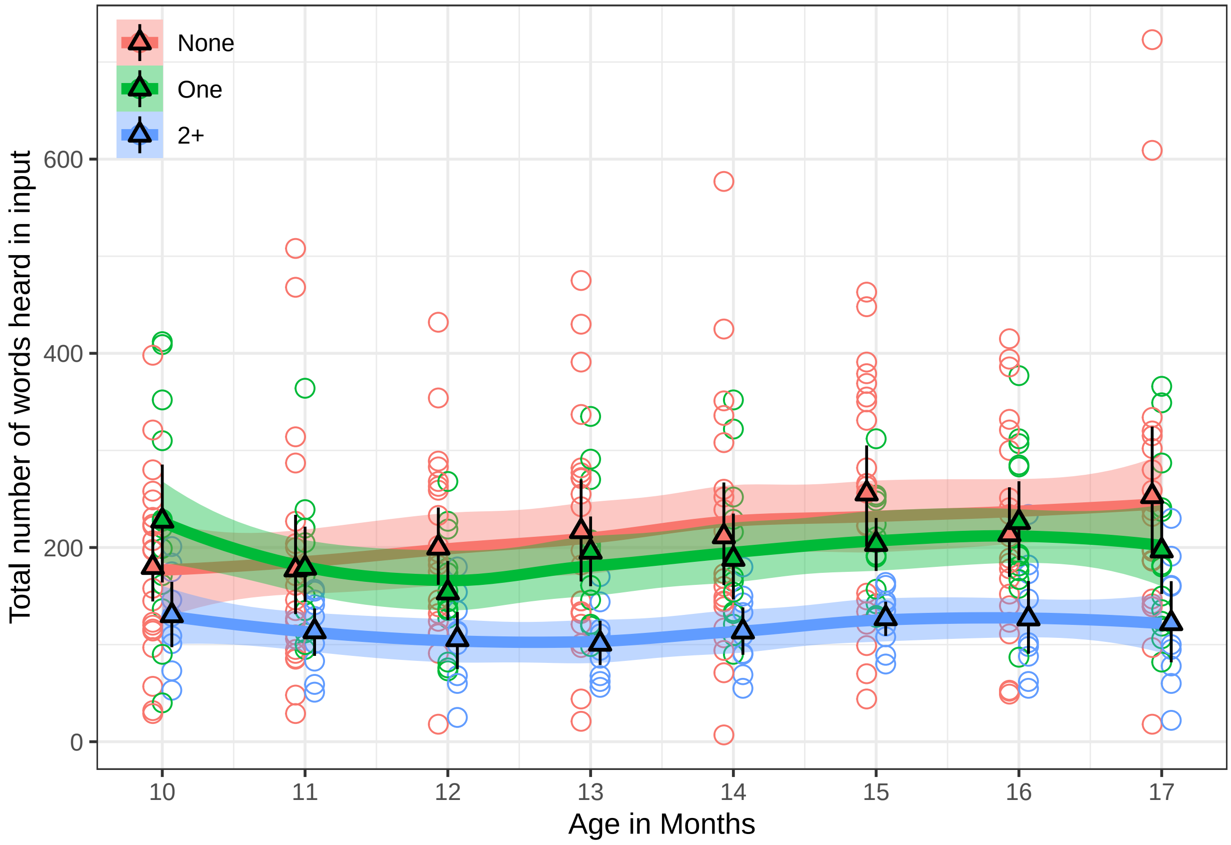

Figure 2. Number of input words produced by caregivers over time (n=43). Colors denote sibling group; line with colored confidence band reflects local estimator (loess) fit over individual infants' input at each month. Triangles indicate mean with bootstrapped CIs computed over each month's data. Points (dodged horizontally for visual clarity) show individual infants' input at each month.

Inspection of both Figures 2 and 3 suggest that both measures were relatively consistent in the data over time, across each sibling group.

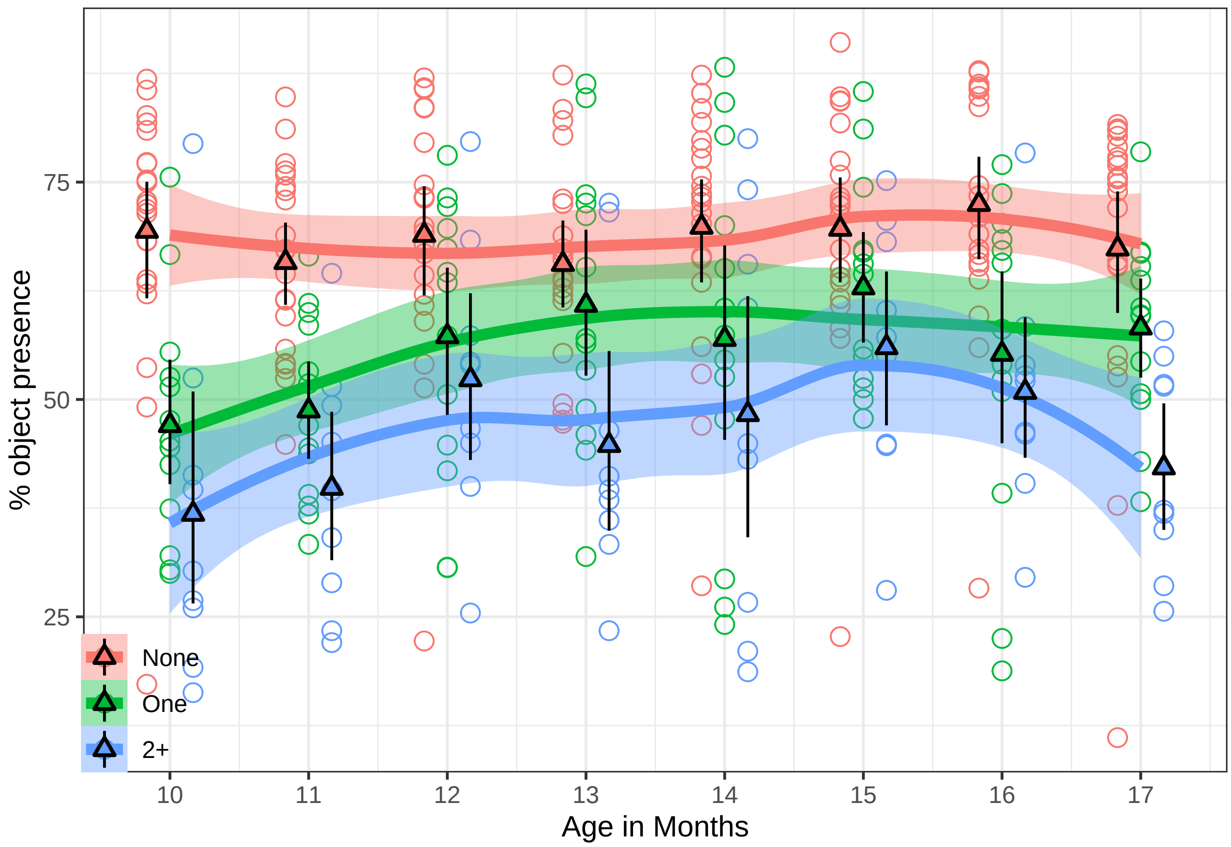

Figure 3. Amount (%) of object presence in infants' input over time (n=43). Colors denote sibling group; line with colored confidence band reflects local estimator (loess) fit over object presence at each month. Triangles indicate mean with bootstrapped CIs computed over each month's data. Points (dodged horizontally for visual clarity) show object presence in individual infants' inputs at each month.

S8: Validating CDI scores with infant productions in the home-recorded data

Parents with more children may have been less aware of the variety of words that their infant could produce, owing to having less time and attention capacity to closely observe their infant's word production. To test the reliability of the reported CDI data used in our analysis, we draw on infants' word production in the home-recorded data (video and audio recordings). Figure 4 shows positive correlations between reported productive vocabulary size (CDI) at 18 months and total number of word types produced by the

infants in the video and audio recordings by 17 months. Outcomes from Pearson correlation tests are shown in Table 10; correlations are positive across all groups, but only significant in the One and None groups, likely owing to the small sample size (n=7) in the 2+ group.

Table 10

Pearson correlation coefficients (r) and p values of correlations between reported productive vocabulary (CDI) at 18 months and number of words produced by infants in the audio/video recordings between 10 and 17 months across sibling groups.

SibGroup	n	r	p value
None	18	0.87	<0.001
One	11	0.92	<0.001
2+	7	0.50	0.259

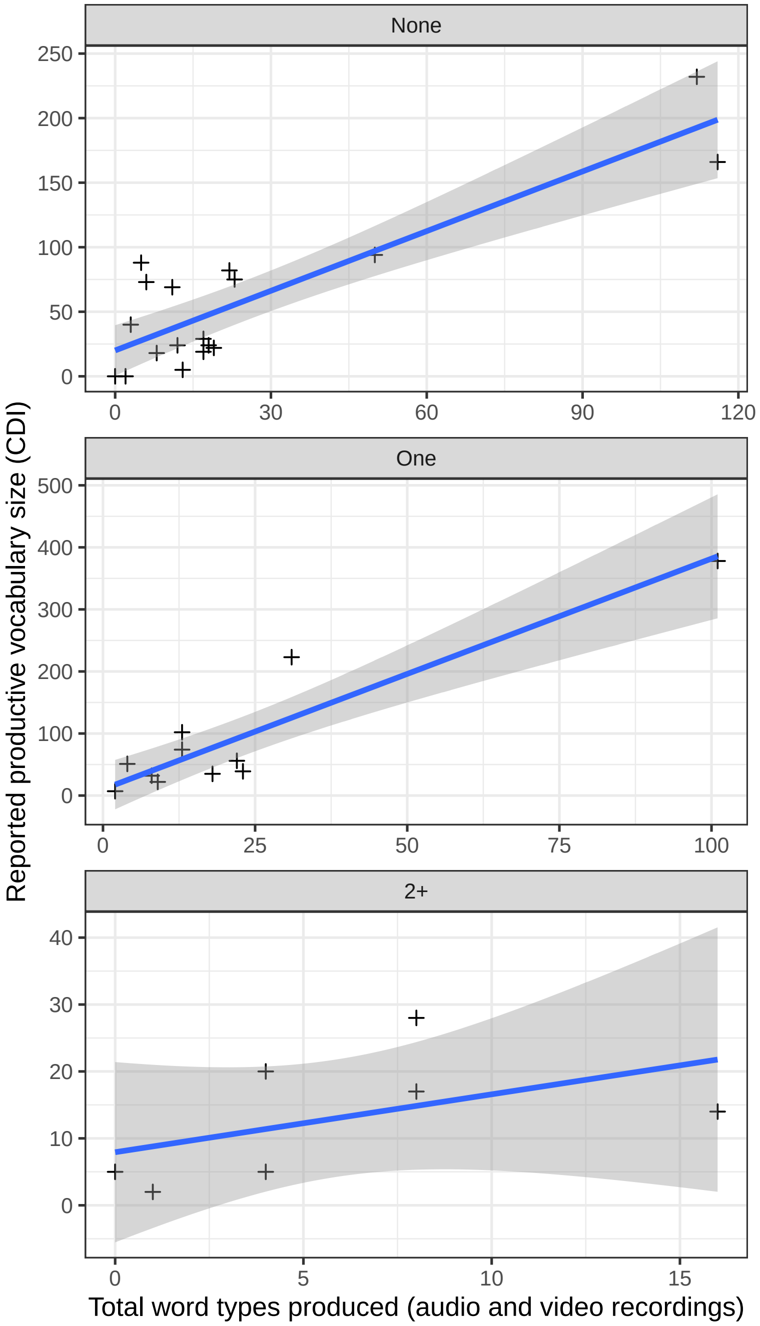

Figure 4. Correlation between reported productive vocabulary at 18 months and n word types produced in the audio and video recordings at 10-17 months (n=43), faceted by sibling group. Crosses represent individual participants in each sibling group, blue lines represent the predicted relationship between the two variables with 95% confident intervals for each group. Due to missing CDIs at 18 months, this includes data from n = 18/21, 11/12, and 7/9 children with 0, 1, and 2+ siblings, respectively.
